# Supplementary material for: Local Tree Diversity Suppresses Foliar Fungal Infestation and Decreases Morphological but Not Molecular Richness in a Young Subtropical Forest
Source: J Fungi (Basel). 2021 Feb 27;7(3):173. doi: 10.3390/jof7030173 (PMC7997179; doi:10.3390/jof7030173)
Supplement: Supplementary file 1 [file jof-07-00173-s001.zip › SM joF_revised.pdf]

Journal of
Fungi

Supplementary Materials

Article Title: Local tree diversity suppresses foliar fungal infestation and decreases morphological but not molecular richness in a young subtropical forest

Authors: Mariem Saadani, Lydia Hönig, Steffen Bien, Michael Koehler, Gemma Rutten, Tesfaye Wubet, Uwe Braun and Helge Bruelheide

The following Supporting Information is available for this article:

Figure S1. OTUs' sequences assigned taxa to genus level.

Figure S2. Relative abundance (%) of functional groups (Guilds) of all samples.

Figure S3. Effect of Shannon diversity on pathogen OTU richness.

Figure S4. Effect of *Liquidambar formosana* proportion on fungal infestation.

Figure S5. Relationship between Mean fungal richness and OTU richness.

Table S1. Detected fungal species/fungal complexes per tree species by visual assessment.

Table S2. List of operational taxonomic units' taxa.

Table S3. Statistical analysis of differences among tree species in Mean fungal richness, mean fungal infestation and OTU richness.

Table S4. Statistical analysis of Non-target proportion in the local neighbourhood effect on fungal richness, fungal infestation and OTU richness.

Methods S1. Bioinformatics pipeline.

Journal of Fungi

MDPI

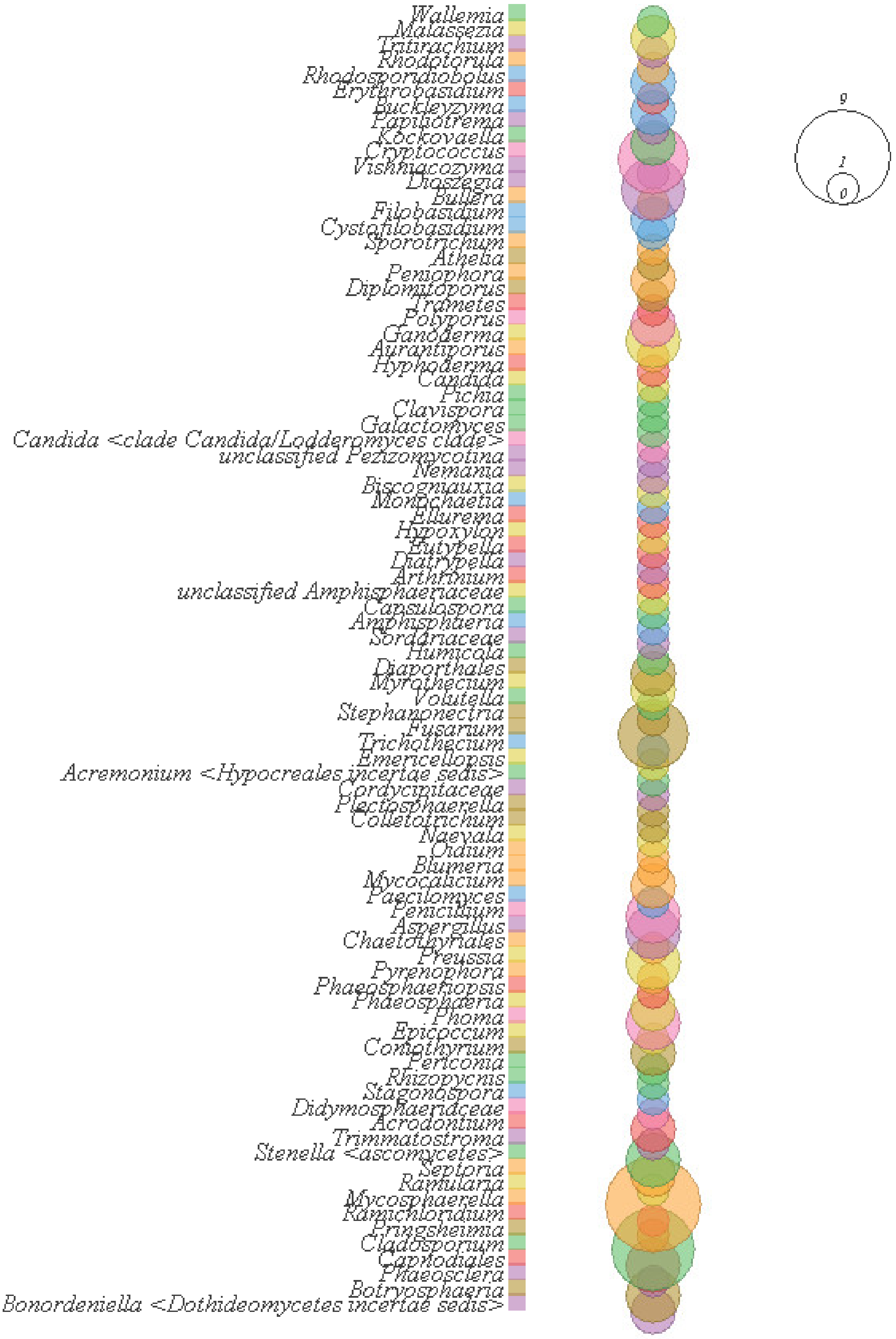

Figure S1. Bubble charts by MEGAN (v6.20.5) for OTUs sequences. Radius of each bubble is proportional to the number of sequences obtained per fungal taxa on genus level.

Journal of Fungi

MDPI

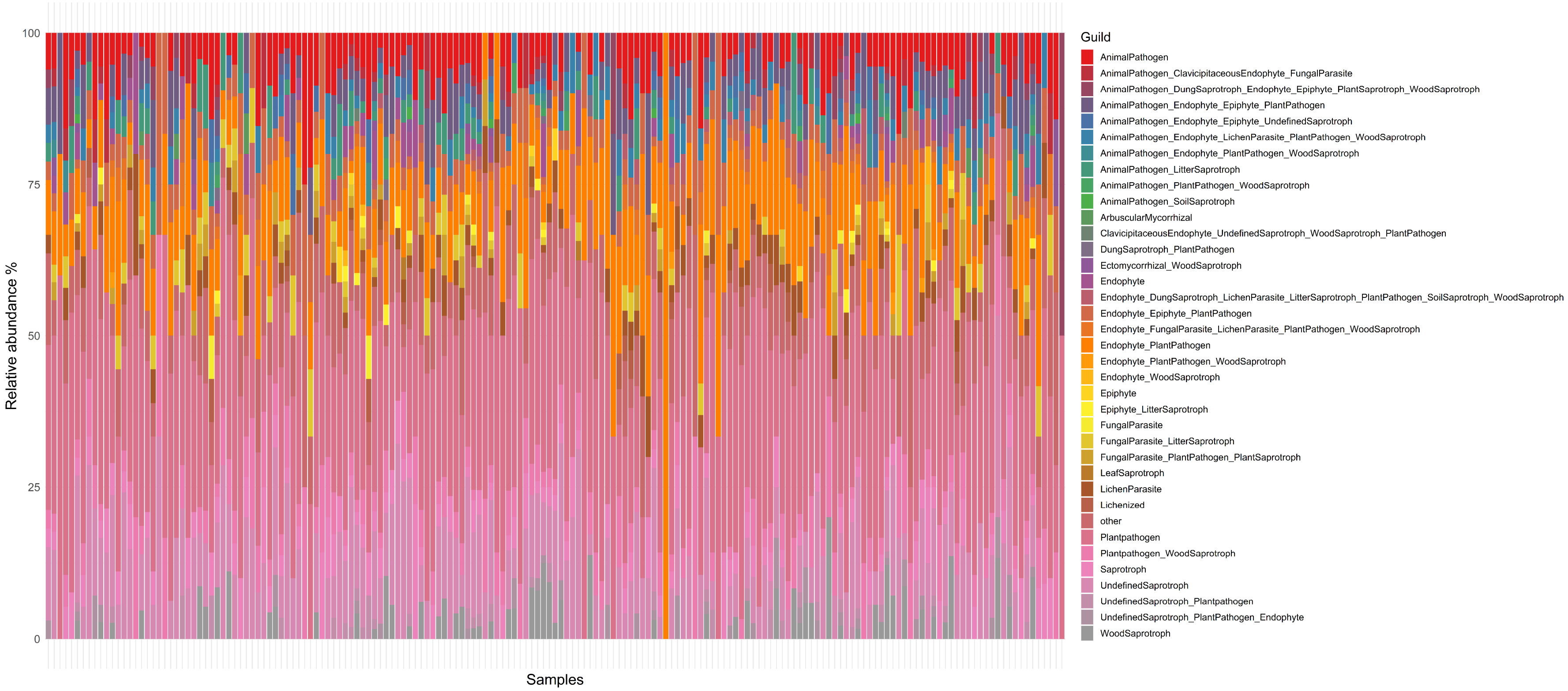

Figure S2. Analysis of fungal community guilds with FUNGuild. The relative abundance (%) for each ecological guild is shown per sample

Journal of Fungi

MDPI

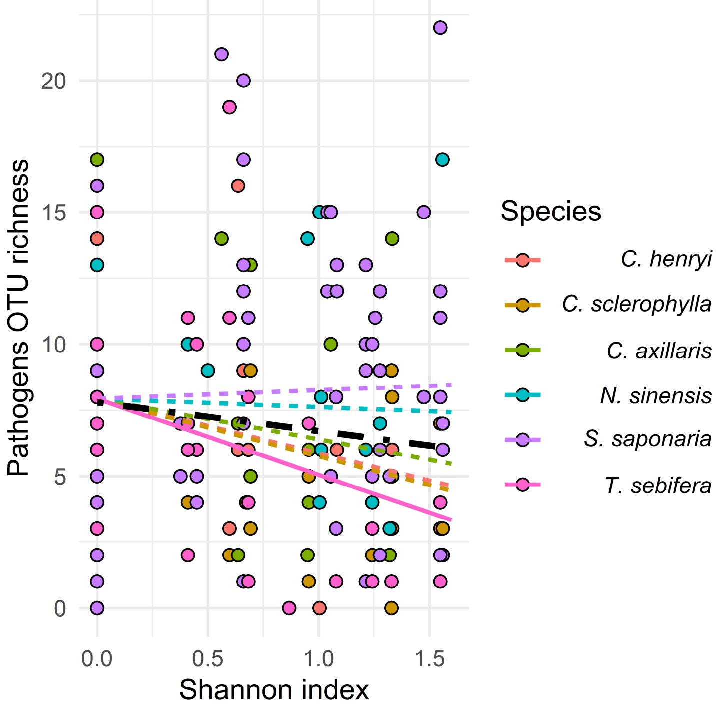

Figure S3. Effect of Shannon diversity in the local neighbourhood on the pathogenic OTU richness ($p = 0.0517$). The overall regression line (in black) was obtained from a mixed model with Shannon diversity as the fixed factor and species identity and neighbourhood composition as random factors. The species-specific regression lines (in colour) were obtained from a mixed model with Shannon diversity and species identity as the fixed factors and neighbourhood composition as random factor. Regression solid, dashed and dash-dotted lines represent significant ($p < 0.05$), marginally significant and non-significant ($p > 0.05$) effects, respectively, according to a non-overlap of the confidence intervals of the unbiased predictors for the slopes with zero.

Journal of Fungi

MDPI

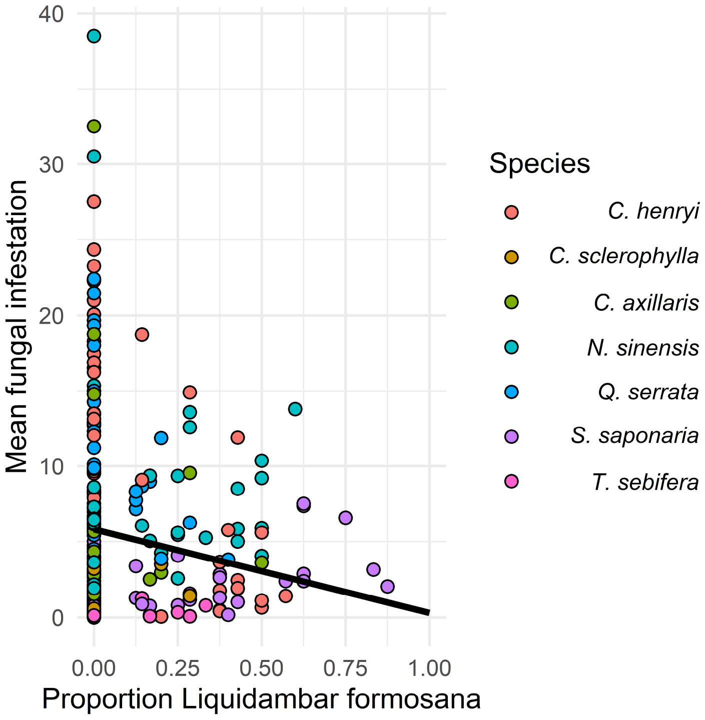

Figure S4. Effect of the proportion of *Liquidambar formosana* (non-target species) in the local neighbourhood on the fungal infestation of all seven other species in the experiment. Regression line was obtained from a mixed effects model with tree species and neighbourhood composition as random factors and it was significant at $p = 0.01089$ (see Table S4).

Journal of Fungi

MDPI

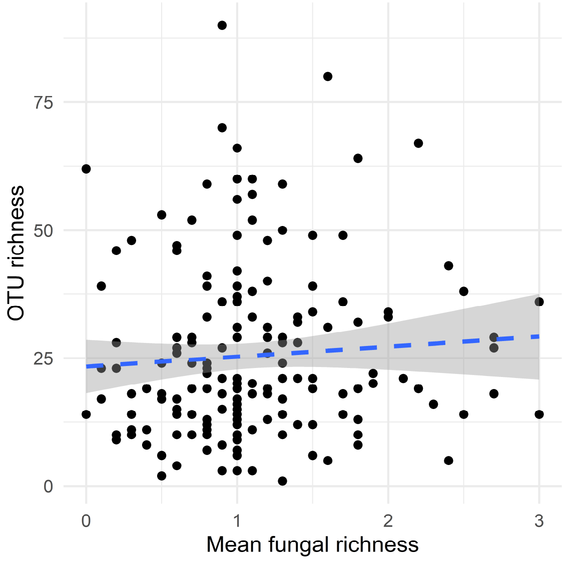

Figure S5. Relationship between fungal richness detected by visual assessment and OTU richness by Next-generation sequencing. The regression line was obtained from a linear model and was non-significant (p = 0.361).

Journal of Fungi

Table S1. Detected fungal species/fungal complexes per tree species by visual assessment, their fungal order/class and their life style under specification of new records (new fungus species, new host, new in China).

(See separate SM Tables excel file (Table S1))

Table S2. List of operational taxonomic unit's taxa identified by Megan/GenBankNCBI/Unit and their ecological guilds by FUNGuild.

(See separate SM Tables excel file (Table S2))

Table S3. Statistical analysis of differences among tree species in Mean fungal richness, mean fungal infestation and OTU richness. Linear mixed effects models included neighbourhood composition as random factor. Significance by ANOVA is indicated by a bold *p* value ($p < 0.05$). The two parameters dfn and dfd refer to the degrees of freedom of the numerator and denominator respectively.

		**Predictor variables**
**Response variables**	**Statistics parameters**	**Tree species**
**a) Mean fungal richness**	dfn	7
	dfd	253.39
	F Value	18.582
	P Value	**< 2.2e-16**
**b) Mean fungal infestation**	dfn	7
	dfd	267.27
	F Value	18.146
	P Value	**< 2.2e-16**
**c) OTU richness**	dfn	5
	dfd	69.32
	F Value	1.586
	P Value	0.1755

Journal of Fungi

MDPI

Table S4. ANOVA table of the effects of the proportion of non-target species in the local neighbourhood on fungal richness, fungal infestation and OTU richness of all the other non-target species (seven each). Mixed effect models were applied with species and neighbourhood composition as random factors. Significant ($p < 0.05$) slope and p values are indicated in bold fonts.

	a) Fungal Richness		b) Fungal Infestation		c) OTU Richness	
**Tree species**	**Estimate**	***P* Value**	**Estimate**	***P* Value**	**Estimate**	***P* Value**
C. henryi	0.1072	0.5408	3.7457	0.0519	9.7129	0.3682
C. sclerophylla	-0.4597	0.1019	-3.6627	0.2082	-15.658	0.3793
C. axillaris	0.1089	0.5556	1.9516	0.3246	13.972	0.1453
L. formosana	-0.2075	0.3092	**-5.5196**	**0.0109**	-0.4839	0.9497
N. sinensis	0.0283	0.9084	-3.4987	0.1119	-1.1071	0.9224
Q. serrata	-0.0921	0.6366	-1.3155	0.5000	-8.9951	0.4376
S. saponaria	0.1547	0.5466	-4.3193	0.1207	-31.573	0.1959
T. sebifera	-0.1186	0.6665	-0.3866	0.8952	-7.2754	0.6101

Journal of
Fungi
MDPI

Methods S1. Bioinformatics pipeline showing step by step filtering and clustering commands.

0#index separated fastq files
1.1#demultiplixing/cutadapt
cutadapt -g Sample1=^(TAG) -g Sample2=^(TAG) -g .... -g SampleX=^(TAG) -G Sample1=^(TAG) -G Sample2=^(TAG) -G .... -G SampleX=^(TAG) -o 1_demux_tag/{name}_R1.fastq -p 1_demux_tag/{name}_R2.fastq Index_x.fastq
applied for all index fastq files based on Tags
1.2#Primer_remover
cutadapt -u n -o SampleX.fastq -i 2_SampleX.fastq
1.3#Pair Concatination
curl -sL git.io/pairfq_lite | perl - makepairs -f SampleX_R1.fastq -r SampleX_R1.fastq -fp 3_synchronisation_R1/SampleX_R1.fastq -rp 3_synchronisation_R2/SampleX_R2.fastq -fs 3_unpaired/SampleX_R1.fastq -rs SampleX_R2.fastq
pear -f SampleX_R1.fastq -r SampleX_R2.fastq -o 4_SampleX.fastq
2#concatinate_files
for f in *; do \
>sed -e "s/\(^@M00.*\) .*$/\1;sample=${f%.*};/" $f \
> >> ../2_concat.fastq; \
> done
3#Quality_filtering 35% score lower than 19
fastq_quality_filter -q 19 -p 35 -i 2_concat.fastq -o 3_filter.fastq
4#convert_to_fasta
$fastq_to_fasta -i 3_filter.fastq -o 4_filter.fasta
5#remove duplicate
$vsearch --derep_fulllength 4_filter.fasta --output 5_dereplication.fasta --sizeout --relabel uniq
6#indel_filtering
$perl -pe '$. > 1 and /^>/ ? print "\n" : chomp' 5_dereplication.fasta > 6_indel.fasta
$sed -n '2~2p' 6_indel.fasta | while read l; do echo ${#l} ; done | sort | uniq -c
==>check size if it is in the required range otherwise filter by size using the next command:
$vsearch --fastx_filter 5_dereplication.fasta --fastq_minlen n --fastq_maxlen n --fastaout 6.1_sized.fasta
#change n with required size
7#Frequency_filtering/denoising
$vsearch --cluster_unoise 5_dereplication.fasta --minsize 4 --unoise_alpha 2 --centroids 7_denoising.fasta
$vsearch --cluster_unoise 6.2_sized.fasta --minsize 4 --unoise_alpha 2 --centroids 7_denoising.fasta
8#Chimera filtering
$vsearch --uchime3_denovo 7_denoising.fasta --nonchimeras 8_nochimera.fasta
9#OTU_delimination
#3% Clustering:
$vsearch --cluster_size 8_nochimera.fasta --relabel OTU --id 0.97 --centroids 9_OTU.fasta --sizein
10#Identifying_OTUs
#Mapping reads to OTUs
#the 3% cluster matched to the filtred Data
$vsearch --usearch_global 4_filter.fasta -db 9_OTU.fasta -id 0.97 -otutabout 10_mapresults_count.tsv
11#Identification Using GenBank
$blastn -db /.../ -query 9_OTU.fasta -outfmt 5 -out out.xml -evalue 0.001 #used later by MEGAN (Software)
$blastn -db /.../ -query 9_OTU.fasta -outfmt 5 -out Blast_results.txt -evalue 0.001 -num_descriptions 5 -num_alignments 5
